# Baseline predictors of response to a group-based speech and communication intervention in Parkinson's disease: A secondary analysis of a randomized controlled trial

**DOI:** 10.1177/1877718X261453191

**Published:** 2026-05-25

**Authors:** Hanna Steurer, Ellika Schalling, Erika Franzén, Joakim Körner Gustafsson, Franziska Albrecht

**Affiliations:** 1 Department of Clinical Science, Intervention and Technology, Division of Speech and Language Pathology, 27106 Karolinska Institutet, Stockholm, Sweden; 2 Research & Development Unit, 83294 Stockholms Sjukhem, Stockholm, Sweden; 3 Department of Public Health and Caring Sciences, Speech-Language Pathology, 8097 Uppsala University, Uppsala, Sweden; 4 Geriatrics, Rehabilitation Medicine and Pain Center, 59561 Uppsala University Hospital, Uppsala, Sweden; 5 Department of Neurobiology, Care Sciences and Society, Division of Physiotherapy, 27106 Karolinska Institutet, Stockholm, Sweden; 6 Women's Health and Allied Health Professionals Theme, Medical Unit Allied Health Professionals, 59562 Karolinska University Hospital, Stockholm, Sweden

**Keywords:** Parkinson's disease, speech intervention, voice, random forest, precision rehabilitation

## Abstract

**Background:**

Speech and voice symptoms are common in Parkinson's disease, yet predictors of response to behavioral speech interventions are unclear.

**Objectives:**

To identify predictors of responsiveness to a speech and communication group-intervention (HiCommunication) and contextualize findings against active controls.

**Methods:**

This secondary analysis of a randomized controlled trial included intervention completers. Responders were defined for voice intensity (increase ≥2 decibels) and voice quality (Acoustic Voice Quality Index decrease ≥0.54). Nineteen baseline clinical, motor, cognitive, perceptual, and acoustic variables were entered into Random Forest classifiers. Primary models excluded the baseline value of the target domain; baseline-including variants were sensitivity analyses. Performance was evaluated using Cohen's kappa, precision, recall, and specificity; key predictors were examined using partial dependence.

**Results:**

In HiCommunication (n = 35), the primary voice intensity model showed moderate agreement (kappa = 0.57; precision = 0.79; recall = 0.90; specificity = 0.64). Higher baseline perceptual ratings of reduced loudness and overall speech deviation, as well as higher Acoustic Voice Quality Index values, were associated with a higher predicted probability of improvement in voice intensity, whereas higher levodopa equivalent daily dose was associated with a lower response probability; postural instability/gait difficulty or tremor-dominant motor phenotypes showed higher response probability than the indeterminate phenotype. Voice quality models were below chance without baseline Acoustic Voice Quality Index but reached kappa 0.38 when included. Active control models showed low performance.

**Conclusions:**

Clinically accessible baseline measures predicted improvement in voice intensity following HiCommunication with moderate accuracy. Perceptual ratings may support expectation-setting and individualized planning of group-based speech intervention in Parkinson's disease, but findings require replication.

ClinicalTrials.gov ID: NCT03213873, doi: 10.1177/1545968321999053

## Plain Language Title

Who benefits most from group-based speech and communication therapy in Parkinson's disease? This study explored whether simple clinical and speech measures collected before treatment can help predict which people with Parkinson's disease are most likely to improve their voice loudness after participating in an intensive group-based speech and communication program.

**Plain Language Summary:**

Speech and communication difficulties are common in Parkinson's disease and can affect everyday interactions and quality of life. Group-based speech and communication therapies can help, but not everyone benefits in the same way. Understanding who is most likely to improve could help clinicians and patients set realistic expectations and plan treatment more effectively.

This study examined whether information collected before treatment could help predict who benefits most from a group-based speech and communication program called HiCommunication. The study used data from a previously completed randomized controlled trial in which people with Parkinson's disease participated either in the speech program or in an active control program focused on balance and gait.

Participants were classified as responders or non-responders based on whether they showed meaningful improvement after treatment in voice loudness or voice quality. The researchers then examined whether baseline characteristics such as clinical features, medication dose, and speech assessments were related to improvement.

The results showed that improvement in voice loudness after HiCommunication could be predicted with moderate accuracy using simple measures commonly collected in clinical practice. People who had more noticeable reduced loudness and overall speech deviation before treatment, as well as poorer voice quality, were more likely to improve their voice loudness. Lower medication dose and certain motor subtypes were also linked to a better response. Changes in voice quality were more difficult to predict, and prediction models did not perform well for participants in the balance training control program.

These findings suggest that basic speech ratings and clinical information collected before treatment may help support conversations about likely benefit from group-based speech therapy in Parkinson's disease. However, the results are preliminary and need to be confirmed in larger studies.

Parkinson's disease (PD) is a heterogeneous neurodegenerative disorder with motor and non-motor manifestations that extend to speech and communication. Hypophonia, reduced prosodic variation, voice quality disturbances, and articulatory imprecision are common and contribute to participation restrictions and decreased quality of life.^1,2^ Growing evidence indicates that targeted speech-language interventions can improve speech and voice symptoms and functional communication in people with PD,^3–8^ potentially through experience-dependent neuroplasticity mechanisms. Principles such as high intensity, repetition, specificity, salience, and progressive task challenge have been proposed to optimize treatment effects.^9^

Our group developed HiCommunication, a 10-week group intervention grounded in these principles and designed to progressively increase cognitive–communicative load while training louder, clearer speech. HiCommunication employs hierarchical training, integrates clinic-based group sessions with at-home practice, and aims to facilitate transfer from structured tasks to everyday communication. Feasibility work demonstrated high acceptability and adherence, and a subsequent randomized controlled trial (RCT) with blinded assessors indicated beneficial effects on speech and voice outcomes as well as effective connectivity changes in a speech-motor brain network relative to an active control.^4,10–12^

Despite encouraging group-level effects, substantial inter-individual variability is typical in PD. Differences in disease phenotype, medication, motor and cognitive status, and baseline speech characteristics can influence responsiveness.^13,14^ Consequently, even effective programs may show attenuated mean effects if subtypes respond differently. Identifying baseline features that characterize responders versus non-responders is therefore critical for precision rehabilitation—to optimize referral and tailor intervention content and progression.

Prognostic studies of speech outcomes in PD have largely focused on surgical contexts (e.g., subthalamic nucleus deep brain stimulation), where baseline speech status and disease characteristics have been linked to postoperative change.^13,15–19^

However, predictors of response to behavioral speech interventions remain largely unexplored, despite their central role in rehabilitation and clinical decision-making. To date, no RCT of speech intervention in PD has systematically examined baseline predictors of individual treatment response. Within rehabilitation research more broadly Albrecht, Johansson^20^ explored responsiveness to highly challenging balance training (HiBalance) using Random Forest (RF) modelling. They classified participants as high, low, or non-responders across balance, gait, and physical activity outcomes, showing that responsiveness can be predicted to some extent from baseline gait and fall history. Together, these studies illustrate the value of responder analyses for precision medicine in PD and provide a rationale for applying similar approaches to speech and communication interventions.

Building on this rationale, the present study leverages data from the Exercise in Parkinson's Disease and Neuroplasticity (EXPANd) trial to investigate determinants of responsiveness to HiCommunication. We examine two clinically meaningful response domains - voice intensity and voice quality - classifying participants as responders or non-responders using criterion-based thresholds. To contextualize speech-specific effects, we conduct parallel responder analyses for the trial's active control intervention, HiBalance, which does not target speech or voice. Baseline variables were selected to capture domains previously implicated in treatment responsiveness and functional heterogeneity in PD. Demographic, clinical, motor, and cognitive measures reflect disease phenotype and capacity for learning, while perceptual and acoustic speech measures index baseline speech impairment and room for improvement. Together, these domains were included to enable a clinically grounded exploration of factors associated with individual response to speech and communication intervention. Our goal was to identify baseline characteristics associated with domain-specific response and generate clinically interpretable insights to guide screening and individualized intervention planning in PD.

## Methods

### Study design and participants

This study was a secondary analysis of data from the randomized controlled trial EXPANd (ClinicalTrials.gov ID: NCT03213873), described in detail in the published protocol^21^ and on the Open Science Framework (OSF; https://osf.io/s952 g/). EXPANd was a parallel-group RCT in which participants were allocated to either a speech-focused intervention (HiCommunication) or an active balance training control (HiBalance). Participants were randomized 1:1 using a computer-generated sequence (random.org) by an independent individual; allocation was concealed in sealed opaque envelopes and assessors were blinded. The present secondary analysis (preregistered on OSF at https://osf.io/75dae) was restricted to intervention completers (≥60% attendance) and therefore does not represent an intention-to-treat comparison. EXPANd was performed in line with the principles of the Declaration of Helsinki and approved by the Regional Ethical Review Board Stockholm (2016/1264-31/4, 2017/1258-32, 2017/2445-32). The participants received written and oral information and provided written informed consent.

Participants were individuals diagnosed with idiopathic PD, meeting the following inclusion criteria: Hoehn and Yahr stages 2–3 (indicating mild-to-moderate disease severity), age ≥60 years, a score of ≥21 on the Montreal Cognitive Assessment (MoCA), ≤27 on the Mini-Balance Evaluation Systems Test, at least one self-reported symptom related to speech, and at least one related to balance. Individuals were excluded if they had comorbidities substantially affecting balance, speech, or voice.

Participants were randomized to one of two 10-week group-based interventions: HiCommunication^10^ or HiBalance.^22^ Both interventions were delivered in small-group settings and adhered to principles of intensity, repetition, and task progression.

Post-intervention assessments were conducted within two weeks after completion of the 10-week intervention period. All baseline predictors were obtained prior to randomization. The MoCA was administered at baseline only and was not repeated post-intervention.

Only participants who were completers of the respective interventions, i.e., participated in at least 60% of the intervention sessions^4,23^ were included in the present study (n = 73). Nine participants in the HiCommunication group and five participants in the active control group had missing values (1.7% and 1.0% out of all the predictor variables, respectively), which we imputed with Random Forest regression (R package “missForest”) (see Supplement).

### Interventions

HiCommunication^10^ spans 10 weeks and involves three weekly sessions led by a speech-language pathologist: two group sessions held in the clinic with six to eight participants, and one home-training session. Each intervention session lasted 60 min. Session attendance (≥60% for inclusion) was used as the operational adherence criterion in this secondary analysis. Home-practice logs were collected in the parent trial but were not modeled here because diary completeness and quantification were not comparable across participants.

The intervention follows a hierarchical design, with cognitive demands gradually increasing over time. Training begins with exercises focusing on producing a louder voice while maintaining good voice quality and clear speech in simple tasks. As the program progresses, participants apply these improved speech techniques during increasingly complex cognitive activities, such as answering progressively challenging questions or engaging in memory and association tasks. Conversation exercises further raise cognitive load and facilitate the transfer of improved speech behaviors to more natural, everyday communication situations. Home training mirrors these principles, but self-directed with participants following a training booklet and logging their practice in a diary.

HiBalance, the active control intervention, consists of sensorimotor balance and gait training targeting motor agility, sensory integration, anticipatory postural adjustments, and limits of stability.^24^ Session structure and frequency match the HiCommunication arm.

### Responder classification

Participants were classified as responders or non-responders based on the change (delta) between pre- and post-intervention values. Responsiveness was assessed in two domains: (I) voice intensity, measured by voice sound level in C-weighted decibels (dBC), and (II) voice quality, measured by the Acoustic Voice Quality Index (AVQI). The thresholds for classification as a responder were:

Voice intensity: An increase of ≥2 dBC, consistent with typical differences in voice sound level between people with PD and matched healthy controls.^4,25^

Voice quality: A reduction of ≥0.54 in AVQI, based on previously established reliable change criteria.^26^ Participants who did not meet these criteria were considered non-responders.

**Figure 1.**
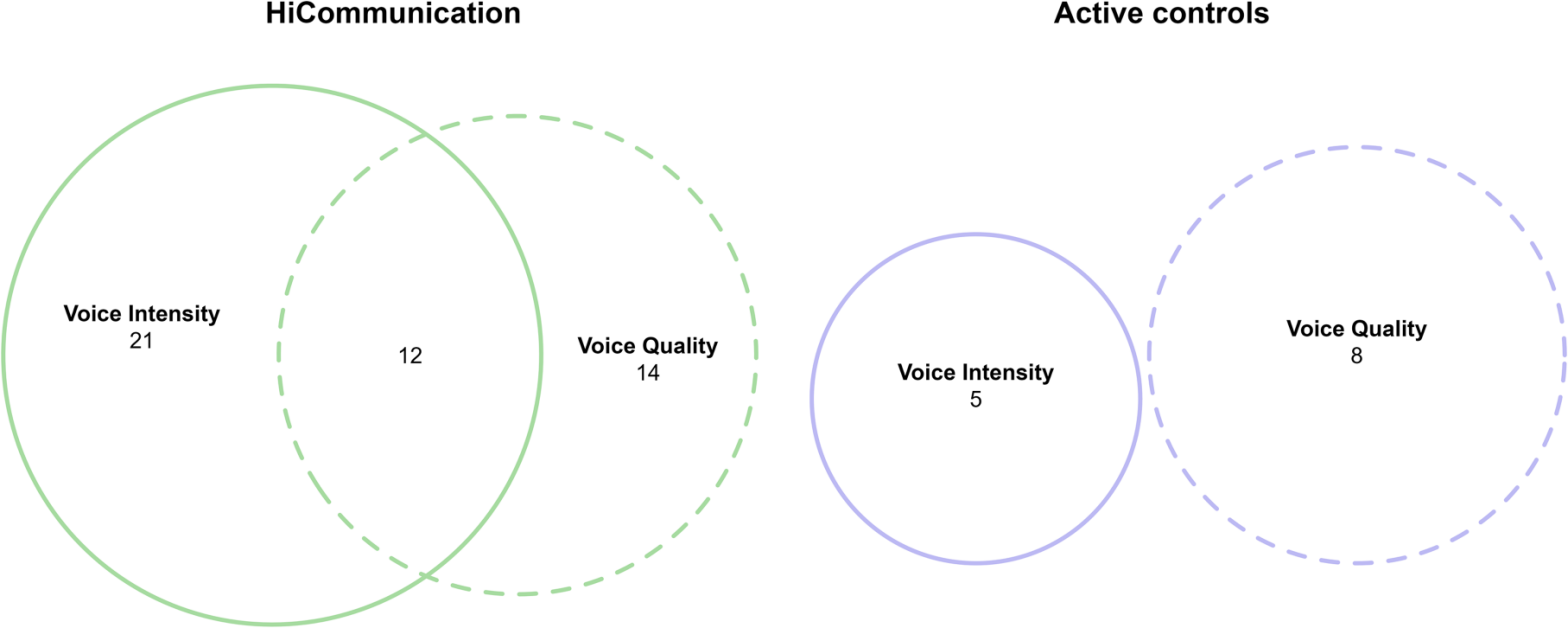
Euler plot of the overlap between the responsiveness levels of responder domains. 304 × 152 mm (300 × 300 DPI).

Importantly, these thresholds are pragmatic rather than anchored to minimal clinically important differences, which are not established for these measures. The ≥2 dB criterion was chosen to represent a potentially perceptible and clinically meaningful shift in vocal intensity in PD relative to typical PD–control differences reported in the literature, and to capture change likely to be relevant for everyday communicative audibility. In contrast, the AVQI threshold represents reliable change beyond measurement error rather than patient-perceived benefit. In the HiCommunication group (n = 35), 21 participants were responders and 14 non-responders in the voice intensity domain (Figure 1, left panel). For voice quality, 14 were responders and 21 non-responders, with 12 participants classified as responders in both domains. In the active control group (n = 38), 5 participants were responders for voice intensity and 8 for voice quality (Figure 1, right panel), with no participants responding in both domains. The low responder proportion in the active control arm provides a contextual benchmark and supports that the responder definition is not trivially met by participation in any group program.

### Random forest supervised classification analysis

RF models were used to explore multivariate patterns of response rather than to test causal effects or derive clinical decision rules. RF constructs multiple decision trees from bootstrapped samples of the data and aggregates predictions across trees using majority voting, which enhances prediction accuracy and reduces overfitting.^27^ At each decision node, a randomly selected subset of predictors is used to determine the optimal split, thereby minimizing inter-tree correlation and increasing model robustness.

RF was selected because it can model complex, nonlinear relationships and interactions between variables, handle both continuous and categorical predictors with minimal preprocessing, and is relatively robust to multicollinearity. It also performs well when the number of predictors is large relative to the number of observations—conditions under which conventional regression approaches, such as logistic regression, are prone to overfitting or convergence problems. In addition, RF provides internal (out-of-bag, OOB) estimates of model performance and permutation-based measures of variable importance, indicating which baseline characteristics most strongly predict individual responsiveness to the intervention.^27–29^ Given our large set of demographic, clinical, motor, perceptual, and acoustic variables, and the expectation of nonlinear response profiles, RF was preferable to linear models.

### Leakage control

As preregistered (OSF: https://osf.io/75dae), we first fit RF models including the baseline value of the outcome domain (voice sound level for voice-intensity; AVQI for voice-quality). Because a domain's baseline can mechanically relate to change-based responder labels, we also fit baseline-excluded variants to estimate the incremental value of other predictors and to avoid interpretive circularity.

### Predictor variables

Nineteen variables, all collected at baseline (pre-intervention), were included as potential predictors in responder classification models (Table 1). Predictors were selected a priori based on literature, clinical relevance, and feasibility within routine clinical practice. In addition to representing motor, perceptual, cognitive, and acoustic domains, variables were chosen to reflect measures that can be readily collected in standard outpatient settings. This approach was intended to enhance translational applicability and support future development of clinically usable screening tools for individualized treatment planning. Data types included continuous measures (e.g., scale scores, counts) and categorical classifications (e.g., cognitive and motor subtypes). Baseline measures were obtained using a combination of clinician-administered assessments (motor, cognitive, dysarthria severity, perceptual speech ratings), participant-reported questionnaires (mood, self-rated speech and communication), wearable-based activity monitoring, and acoustic analyses of standardized speech recordings. Clinician-rated measures were completed by trained speech-language pathologists or physiotherapists blinded to group allocation.

**Table 1.** Baseline variables included in responder classification models.

Variable(s)	Description / measurement	Data type	Source
Age	Participant age in years	Continuous	Demographic form
Levodopa Equivalent Daily Dose (LEDD)	Total daily dose of dopaminergic medication, calculated as LEDD	Continuous	Clinical record
Motor severity (MDS-UPDRS Total)	Motor examination score from the Movement Disorder Society Unified Parkinson's Disease Rating Scale^30^	Continuous	Clinician-rated scale
Cognitive function	Montreal Cognitive Assessment (MoCA); global cognitive screening tool^31^	Continuous	Clinician-administered test
Anxiety (HADS-Anxiety)	Anxiety subscale from the Hospital Anxiety and Depression Scale^32^	Continuous	Self-report questionnaire
Depression (HADS-Depression)	Depression subscale from the Hospital Anxiety and Depression Scale^32^	Continuous	Self-report questionnaire
Daily physical activity (steps/day)	Average daily step count measured using the ActiGraph GT3X + accelerometer worn on the hip for seven consecutive days	Continuous	Wearable device
Cognitive subtype	Classification of cognitive status based on^33^ criteria	Categorical	Derived from test scores
Motor subtype	Classification of motor phenotype based on Stebbins, Goetz^34^ criteria	Categorical	Derived from MDS-UPDRS
Dysarthria severity	Overall severity rating from the Swedish Dysarthria Assessment^35^	Continuous (bounded scale 0.0 to 3.0)	Clinician-administered test
Perceptual speech ratings	Three individual clinician-rated speech characteristics: general impression of speech deviation, reduced loudness, and harshness	Continuous on a visual analogue scale (VAS) from 0-1000, higher scores indicated more severe perceived speech deviation (3 variables)	Clinician-rated scale
Acoustic measures of diadochokinesis	Two acoustic measures of diadochokinesis (DDK): alternating motion rate (AMR) and sequential motion rate (SMR)	Continuous (2 variables)	Acoustic analysis
Verbal fluency	Two verbal fluency tasks: phonological and semantic verbal fluency was assessed using a Swedish adaptation of standard verbal fluency tasks^36^	Continuous (2 variables)	Clinician-administered test
Self-rated speech and communication changes^35^	Questionnaire on Acquired Speech Disorders (QASD)^35^	Continuous (bounded scale 0.0 to 3.0)	Self-report questionnaire
Voice sound level	C-weighted average decibel (dB) during text reading	Continuous	Acoustic analysis using Sopran (version 1.0.22 Tolvan Data)
Dysphonia	Acoustic Voice Quality Index (AVQI), a composite measure of dysphonia based on sustained vowel and connected speech^37^	Continuous	Acoustic analysis using AVQI software (version 01.03, Phonanium, 2021)

Note: Some rows represent multiple individual predictors.

### Classified variables

Cognitive and motor subtypes were assigned using established classification systems. Cognitive subtypes followed Litvan, Goldman,^33^ categorizing participants based on neuropsychological test profiles into normal cognition or mild cognitive impairment (MCI). Motor subtypes were classified into tremor-dominant (TD), postural instability/gait difficulty (PIGD), or indeterminate (IND) phenotypes using MDS-UPDRS III scores following Stebbins, Goetz.^34^ Tremor and PIGD subscores were calculated from the corresponding MDS-UPDRS item sets (tremor: items 2.10 and 3.15–3.18; PIGD: items 2.12–2.13 and 3.10–3.12). Participants were classified as TD when the tremor/PIGD ratio was ≥1.15, as PIGD when the ratio was ≤0.90, and as indeterminate otherwise.

### Acoustic and auditory-perceptual ratings of speech recordings

Auditory-perceptual speech ratings and acoustic measures were derived from standardized speech recordings collected before and after the intervention in a sound-proof recording studio. Recordings were obtained using a head-mounted microphone positioned at a fixed mouth-to-microphone distance (15 cm) to minimize variability in voice sound level related to microphone placement. Recordings were performed according to a standardized protocol including sustained vowel phonation and connected speech (text reading), and the same recording setup was used across participants. Details on acoustic analyses^4^ and auditory-perceptual analyses^11^ have been previously reported. Speech intelligibility was assessed in the parent trial using the Swedish Test of Intelligibility (words and sentences). However, baseline scores demonstrated a ceiling effect, with the majority of participants performing at or near maximum levels, reflecting generally mild dysarthria in the cohort. Consequently, intelligibility was not included in the responder classification models, as limited variability reduces predictive utility.

### Statistical analysis

All statistical analyses were conducted using R (version 4.4.2). Baseline demographic, clinical, motor, and speech variables were compared between responders and non-responders using non-parametric Mann–Whitney U tests for continuous variables and chi-squared tests for categorical variables, as appropriate. These baseline comparisons were descriptive and exploratory and were not used to select predictors for the RF models.

RF classification analyses were conducted using the randomForest package (version 4.7-1.2).^28^ Four separate classification models were developed to examine differential response patterns depending on intervention type and outcome domain: response in voice intensity and voice quality following HiCommunication, and response in voice intensity and voice quality following the active control intervention HiBalance. RF models were fit with 20,000 trees and terminal node size 7. Based on hyperparameter checks (Supplement), the number of variables randomly selected at each split (mtry) was set to 4 or 5 depending on the model.

### Model evaluation

Because the proportion of responders and non-responder differed across datasets, we compared model accuracy to naïve baselines defined by class prevalence. A classifier that always predicts the majority class achieves an accuracy equal to that class's proportion (“chance-level” accuracy). In HiCommunication–voice intensity, 21/35 (60%) were responders and 14/35 (40%) non-responders; thus, a trivial “all-responder” rule would score 60% accuracy. In the active-control–voice intensity set, only 5/38 (13%) were responders (87% non-responders), so an “all–non-responder” rule would already reach 87% accuracy. Analogous baselines were 60%/ 40% (non-responder/responder) for HiCommunication - voice quality and 79%/ 21% for active-control - voice quality. We therefore report Cohen's kappa (κ) (agreement beyond chance) together with per-class precision, recall (sensitivity), and specificity to provide a fair assessment beyond these naïve baselines. From a clinical screening perspective, recall/sensitivity for responders is particularly relevant because missed responders (false negatives) may represent lost opportunities for effective intervention.

To account for chance agreement, we computed unweighted Cohen's κ based on out-of-bag (OOB) predictions. We report precision (the proportion of predicted positives that are true positives), recall/sensitivity (the proportion of actual positives correctly identified), and specificity (the true-negative rate). Finally, we include Youden's index (sensitivity + specificity − 1) as a single summary of class separation, where 1 indicates perfect discrimination and 0 indicates performance no better than chance. All performance metrics were derived from OOB predictions; no additional cross-validation was performed.

### Variable importance and interpretation

Variable importance was assessed using mean decrease in accuracy, a permutation-based metric.^29^ This approach quantifies the increase in classification error when the values of a variable are randomly permuted. Variables with higher mean decrease accuracy values were considered more predictive.

Because permutation importance can be influenced by correlated predictors, importance rankings were interpreted cautiously and descriptively rather than as reflecting independent causal contribution.

To interpret the marginal effects of these key predictors, partial dependence plots were generated, illustrating the model's predicted probability across the range of a given variable, while holding all other variables constant. Partial dependence plots illustrate marginal associations and do not imply causality. Limitations of partial dependence plots, including potential masking of interactions and the impact of correlated predictors, are acknowledged.

## Results

### Characteristics of responders and non-responders

In the HiCommunication group, significant differences between responders and non-responders in the voice intensity domain were found for baseline voice sound level, levodopa equivalent daily dosage (LEDD), and perceptual ratings of general speech impression of speech deviation and reduced loudness (Mann–Whitney U tests), as well as for motor subtype (Chi-squared test; see Table 2). Responders had lower baseline voice sound levels and lower LEDD compared to non-responders, along with higher perceptual ratings of general speech impression and reduced loudness. Regarding motor subtype distribution, the postural instability and gait disorder subtype was most common in both groups. Among responders, the tremor-dominant subtype was the second most common, whereas the indeterminate subtype was more frequent among non-responders. No significant differences were observed between groups for any other variables.

**Table 2.** Demographics of the participants in the HiCommunication intervention group divided into voice intensity responders and -non-responders.

Variable	NR (n = 14)	R (n = 21)	Total (n = 35)	p-value
Age, years				0.625
Median	71.50	70.00	70.00	
IQR	12.25	7.00	8.50	
Sex, n (%)				0.656
Male	9 (64.3%)	15 (71.4%)	24 (68.6%)	
Female	5 (35.7%)	6 (28.6%)	11 (31.4%)	
Cognitive subtype, n (%)				0.234
Non-MCI	8 (57.1%)	16 (76.2%)	24 (68.6%)	
MCI	6 (42.9%)	5 (23.8%)	11 (31.4%)	
Motor subtype, n (%)				0.040
IND	5 (35.7%)	1 (4.8%)	6 (17.1%)	
PIGD	7 (50.0%)	12 (57.1%)	19 (54.3%)	
TD	2 (14.3%)	8 (38.1%)	10 (28.6%)	
LEDD, mg				0.030
Median	500.00	350.00	405.00	
IQR	205.00	305.00	275.00	
MDS-UPDRS Total				0.674
Median	50.00	51.00	51.00	
IQR	19.75	24.00	23.50	
Steps per day				0.495
Median	6002.50	5949.25	5949.25	
IQR	4657.02	4761.39	4367.20	
Dysarthria test total score				0.189
Median	0.14	0.08	0.11	
IQR	0.24	0.09	0.13	
Questionnaire of Acquired Speech Disorders				0.866
Median	0.29	0.39	0.36	
IQR	0.62	0.39	0.50	
Voice sound level				0.031
Median	72.22	70.06	70.29	
IQR	5.36	4.62	5.30	
Voice sound level Δ				<0.001
Median	−0.84	3.60	2.47	
IQR	2.81	2.33	3.72	
AVQI				0.066
Median	3.21	4.04	3.85	
IQR	1.52	1.68	1.69	
DDK AMR				0.266
Median	6.16	6.20	6.19	
IQR	0.78	1.00	0.92	
DDK SMR				0.920
Median	5.45	5.00	5.20	
IQR	0.89	1.63	1.47	
Perceptual general impression of speech deviation				0.031
Median	182.50	310.00	237.00	
IQR	94.40	152.00	169.50	
Perceptual reduced loudness				<0.001
Median	0.00	257.00	208.00	
IQR	25.75	181.00	361.00	
Perceptual harshness				0.972
Median	120.50	33.00	112.00	
IQR	278.75	171.00	276.50	
HADS Anxiety				0.671
Median	4.50	5.00	5.00	
IQR	2.00	5.00	4.00	
HADS Depression				0.664
Median	3.00	3.00	3.00	
IQR	3.50	2.00	2.50	
Verbal fluency phonological				0.469
Median	37.50	42.00	42.00	
IQR	17.75	24.00	22.50	
Verbal fluency semantic				0.245
Median	33.00	42.00	37.00	
IQR	16.50	13.00	14.50	
Presence intervention, %				0.811
Median	87.50	90.00	90.00	
IQR	8.75	10.00	10.00	

Note: Values are median (IQR) unless otherwise indicated. p-values are unadjusted (Mann–Whitney U for continuous variables; χ^2^ for categorical variables). All variables were derived at baseline unless labelled with Δ (= post – pre). Abbreviations and variable definitions: AVQI, Acoustic Voice Quality Index (0–10; higher = worse dysphonia); DDK-AMR, Diadochokinetic Alternating Motion Rate (/pa/), syllables/s; DDK-SMR, Diadochokinetic Sequential Motion Rate (/pa-ta-ka/), syllables/s; HADS, Hospital Anxiety and Depression Scale subscales (0–21; higher = worse); IND, indeterminate motor subtype (per Stebbins et al.); IQR, interquartile range; LEDD, levodopa equivalent daily dose (mg); MCI, mild cognitive impairment (per Litvan, Goldman^33^); MDS-UPDRS, Movement Disorder Society Unified Parkinson's Disease Rating Scale (higher = worse); NR, non-responder; PIGD, postural instability and gait disorder motor subtype; R, responder; TD, tremor-dominant subtype; Voice sound level, C-weighted SPL during text reading (dBC; higher = louder); Perceptual ratings (general impression of speech deviation, reduced loudness, harshness), clinician VAS 0–1000 (higher = more deviant/greater impairment); Steps per day, 7-day hip-worn accelerometry average; Presence intervention, percentage of attended sessions.

In the voice quality domain, a significant difference between responders and non-responders was observed for AVQI scores, with responders having higher baseline AVQI values than non-responders (Table 3). In addition, significant differences were found for perceptual reduced loudness and DDK SMR.

**Table 3.** Demographics of the participants in the HiCommunication intervention group divided into voice quality responders and -non-responders.

Variable	NR (n = 21)	R (n = 14)	Total (n = 35)	p-value
Age, years				0.649
Median	72.00	69.50	70.00	
IQR	9.00	4.75	8.50	
Sex, n (%)				0.298
Male	13 (61.9%)	11 (78.6%)	24 (68.6%)	
Female	8 (38.1%)	3 (21.4%)	11 (31.4%)	
Cognitive subtype, n (%)				0.656
Non-MCI	15 (71.4%)	9 (64.3%)	24 (68.6%)	
MCI	6 (28.6%)	5 (35.7%)	11 (31.4%)	
Motor subtype, n (%)				0.930
IND	4 (19.0%)	2 (14.3%)	6 (17.1%)	
PIGD	11 (52.4%)	8 (57.1%)	19 (54.3%)	
TD	6 (28.6%)	4 (28.6%)	10 (28.6%)	
LEDD, mg				0.274
Median	450.00	386.50	405.00	
IQR	250.00	260.00	275.00	
MDS-UPDRS Total				0.238
Median	52.00	39.50	51.00	
IQR	22.00	25.50	23.50	
Steps per day				0.727
Median	5775.14	6589.14	5949.25	
IQR	4807.54	4672.36	4367.20	
Dysarthria test total score				0.363
Median	0.13	0.09	0.11	
IQR	0.18	0.09	0.13	
Questionnaire of Acquired Speech Disorders				0.613
Median	0.36	0.34	0.36	
IQR	0.56	0.33	0.50	
Voice sound level				0.377
Median	71.06	70.21	70.29	
IQR	6.05	5.75	5.30	
AVQI Δ				<0.001
Median	0.11	−1.55	−0.44	
IQR	0.96	0.68	1.36	
AVQI				<0.001
Median	3.41	5.08	3.85	
IQR	0.83	0.97	1.69	
DDK AMR				0.613
Median	6.20	6.19	6.19	
IQR	0.55	0.96	0.92	
DDK SMR				0.047
Median	5.60	4.76	5.20	
IQR	1.31	0.94	1.47	
Perceptual general impression of speech deviation				0.342
Median	215.00	300.50	237.00	
IQR	160.00	202.25	169.50	
Perceptual reduced loudness				0.049
Median	26.00	248.00	208.00	
IQR	283.00	209.25	361.00	
Perceptual harshness				0.887
Median	112.00	81.00	112.00	
IQR	281.00	154.25	276.50	
HADS Anxiety				0.932
Median	5.00	5.00	5.00	
IQR	4.00	3.25	4.00	
HADS Depression				0.835
Median	3.00	3.00	3.00	
IQR	4.00	2.00	2.50	
Verbal fluency phonological				0.775
Median	41.00	43.00	42.00	
IQR	22.00	19.50	22.50	
Verbal fluency semantic				0.736
Median	39.00	35.50	37.00	
IQR	17.00	12.50	14.50	
Presence intervention, %				0.932
Median	85.00	90.00	90.00	
IQR	10.00	8.75	10.00	

Note: Values are median (IQR) unless otherwise indicated. p-values are unadjusted (Mann–Whitney U for continuous variables; χ^2^ for categorical variables). All variables were derived at baseline unless labelled with Δ (= post – pre). Abbreviations and variable definitions: AVQI, Acoustic Voice Quality Index (0–10; higher = worse dysphonia); DDK-AMR, Diadochokinetic Alternating Motion Rate (/pa/), syllables/s; DDK-SMR, Diadochokinetic Sequential Motion Rate (/pa-ta-ka/), syllables/s; HADS, Hospital Anxiety and Depression Scale subscales (0–21; higher = worse); IND, indeterminate motor subtype (per Stebbins et al.); IQR, interquartile range; LEDD, levodopa equivalent daily dose (mg); MCI, mild cognitive impairment (per Litvan, Goldman^33^); MDS-UPDRS, Movement Disorder Society Unified Parkinson's Disease Rating Scale (higher = worse); NR, non-responder; PIGD, postural instability and gait disorder motor subtype; R, responder; TD, tremor-dominant subtype; Voice sound level, C-weighted SPL during text reading (dBC; higher = louder); Perceptual ratings (general impression of speech deviation, reduced loudness, harshness), clinician VAS 0–1000 (higher = more deviant/greater impairment); Steps per day, 7-day hip-worn accelerometry average; Presence intervention, percentage of attended sessions.

In the active control group, no significant differences were found between responders and non-responders for any measured variables (Supplementary Tables 1 and 2).

### Model selection and interpretation

Consistent with our preregistration, we built models both with and without the baseline value of the outcome domain (e.g., voice sound level for voice intensity; AVQI for voice quality). To minimize circularity and maintain cross-domain comparability, we treat baseline-excluded models as primary and report them in the main text. Under this specification, only the HiCommunication voice intensity model showed robust, interpretable performance (Table 4).

**Table 4.** Prediction metrics for classification of intervention response using different response domains. The table summarizes confusion matrix values and derived metrics including Cohen's Kappa, precision, recall (sensitivity), specificity, and Youden's index for each model. These metrics reflect the agreement between predicted and observed intervention response (responder vs. non-responder).

Domain	Group	TN	FP	FN	TP	Precision	Recall	Specificity	Youden's index	Cohen's kappa	Class. error
Voice Intensity	HC	9	5	2	19	0.79	0.90	0.64	0.55	0.57	0.20
Voice Intensity	AC	33	0	5	0	NA*	0.00	1.00	0.00	0.00	0.13
Voice Quality	HC	14	7	11	3	0.30	0.21	0.67	−0.12	−0.13	0.51
Voice Quality	AC	29	1	8	0	0.00	0.00	0.97	−0.03	−0.05	0.24

Note: Precision is undefined (NA) for the active control voice intensity model because no true positives (TP) were predicted. All Cohen's Kappa values are unweighted. According to Cohen's guidelines, values of kappa ≤ 0 indicate no agreement, values between 0.01 and 0.20 indicate slight agreement, 0.21–0.40 fair agreement, 0.41–0.60 moderate agreement, 0.61–0.80 substantial agreement, and 0.81–1.00 almost perfect agreement. Classification (Class.) error is calculated as (FP + FN) divided by the total number of observations (TN + FP + FN + TP), representing the overall proportion of incorrect predictions. Abbreviations: HC = HiCommunciation, AC = Active controls, NR = non-responder, R = responder, TN = true negative, FP = false positive, FN = false negative, TP = true positive, NA = not available.

For completeness, we also fit baseline-including variants. In HiCommunication voice intensity, including baseline voice sound level produced a very similar κ and ranking; detailed metrics are provided in Supplementary Table S3 and variable importance (numeric mean decrease accuracy) in Supplementary Table S4. In the voice quality domain, the baseline-excluded model performed below chance (κ < 0), whereas including baseline AVQI improved κ to 0.38 with AVQI dominating importance; we therefore present this as a sensitivity analysis only (detailed metrics in Supplementary Table S3 and variable importance in Supplementary Table S5). In the active-control arm, all models performed at or below chance (κ ≤ 0; Table 4 for primary active control models and Supplementary Table S3 for models including baseline voice sound level/AVQI).

### Variable importance and partial dependence

**Figure 2.**
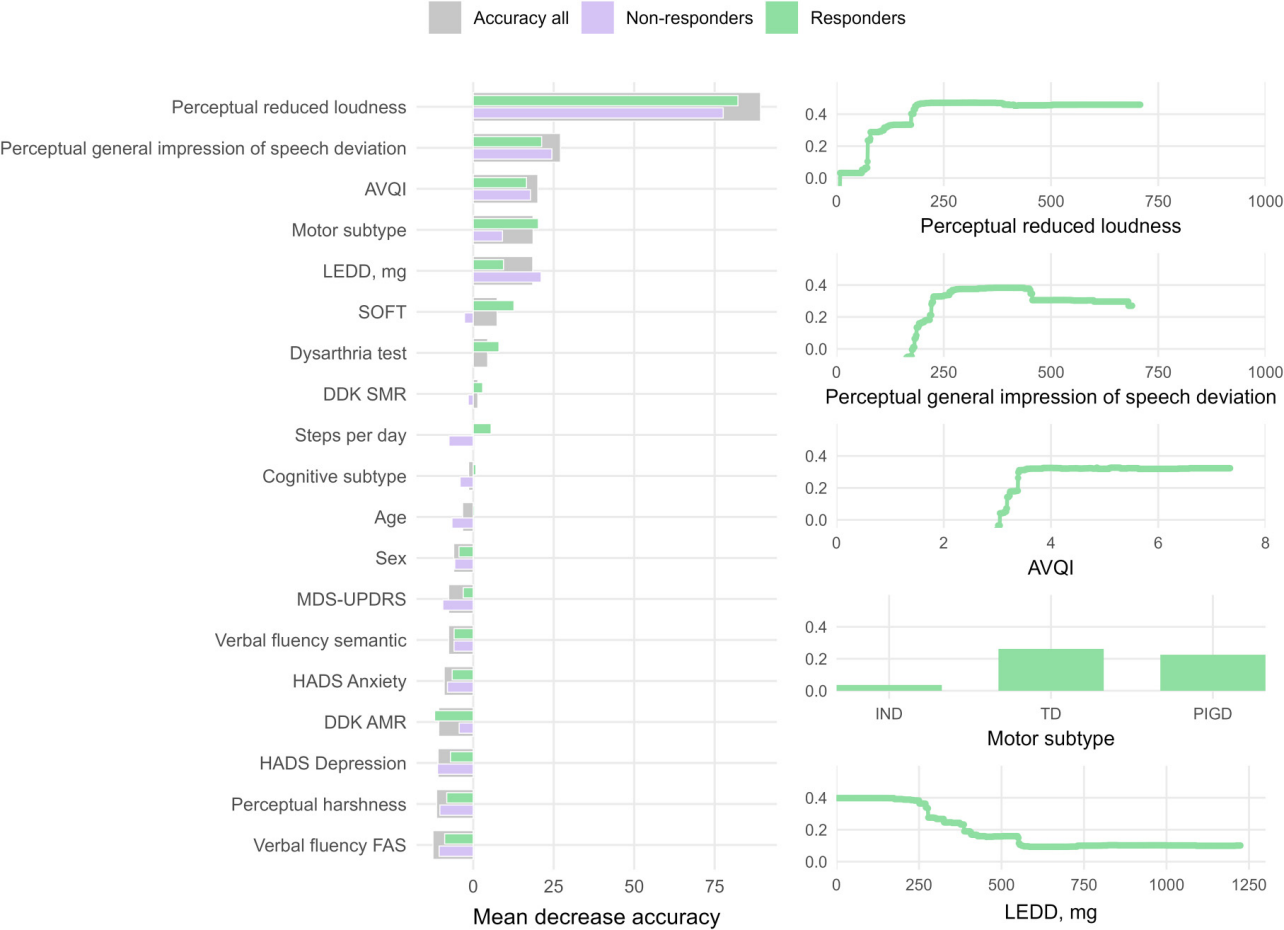
Variable importance. Figure 2 Note. Left panel: Mean decrease in the Random Forest prediction accuracy for each variable used for the decision trees. The decreased accuracy is shown as a percentage increase in the misclassification rate as compared to the out-of-bag rate. Classification probability for each responsiveness level is shown as a function of predicted values of classification variables: Right panel: Partial dependence on the perceptual reduced loudness (visual analogue scale) the perceptual general impression of speech deviation (visual analogue scale), the Acoustic Voice Quality Index (AVQI, score), motor subtype, Levodopa Equivalent Daily Dosage (LEDD, mg) before the intervention. Abbreviations: HADS, Hospital Anxiety and Depression scale; MDS-UPDRS, Movement Disorder Society-sponsored Revision of the Unified Parkinson's disease Rating Scale; DDK-SMR, Diadochokinetic Sequential Motion Rate; DDK-AMR, Diadochokinetic Alternating Motion Rate; QASD, Questionnaire on Acquired Speech Disorders; IND, indeterminate; TD,tremor-dominant; PIGD, postural instability and gait disorder. 233 × 172 mm (300 × 300 DPI).

Results below refer only to the primary HiCommunication voice intensity model (baseline voice sound level excluded). Permutation importance (mean decrease accuracy) quantified how much each predictor contributed to classification (Figure 2, left). The hierarchy was steep: reduced loudness and general impression of speech deviation dominated, followed - at an “elbow” - by AVQI, motor subtype, and LEDD; remaining variables clustered near zero, indicating limited unique signal. This distribution motivated focusing the interpretive plots on the top five predictors.

Partial dependence plots (Figure 2, right) illustrate the marginal effect of each key predictor on the probability of being classified as a responder (*p*Responder). Because the model is binary, the non-responder curve is simply 1 − *p*Responder and adds no new information. Partial dependence plots suggested threshold-like effects: *p*Responder rose to ∼220 (reduced loudness) and ∼200 (general impression of speech deviation) then plateaued; AVQI showed a change near ∼3.5; PIGD/TD > IND for motor subtype; and *p*Responder declined above ∼250 mg LEDD. These effects are marginal (holding other variables at their observed distribution) and should not be interpreted causally; mean decrease accuracy and partial dependence plots can be attenuated by correlated predictors and differences in variable scale/cardinality.

## Discussion

### Summary of findings

In this responder analysis of the EXPANd RCT, baseline perceptual speech ratings, AVQI, motor subtype, and dopaminergic dose emerged as the strongest predictors of benefit from HiCommunication in the voice intensity domain. An RF model that excluded baseline domain measures to avoid circularity classified responders with moderate agreement (κ = 0.57), high recall for responders (0.90), and acceptable precision (0.79). Variable importance and partial dependence indicated threshold-like effects: increasing baseline reduced loudness and general impression of speech deviation were associated with higher response probability up to approximately 220 and 200 VAS units, respectively, after which effects plateaued. AVQI showed a change point around ∼3.5, above which response probability increased. Postural instability and gait disorder and tremor-dominant motor subtypes had higher response likelihood than the indeterminate subtype, and response probability diminished above ∼250 mg LEDD. By contrast, voice-quality models were informative only when baseline AVQI was included, and both models in the active-control group performed at chance, consistent with the absence of targeted speech intervention and few responders.

Baseline group comparisons (Tables 2–3) broadly mirrored the model: voice intensity responders entered with lower voice sound level, higher perceptual scores on reduced loudness and general impression of speech deviation, lower LEDD, and fewer indeterminate motor phenotypes; for voice quality, AVQI differed as well as perceptual reduced loudness and DDK SMR. These univariate contrasts are exploratory and unadjusted, and some derive from the same domain as the responder definition (e.g., AVQI), so they should be viewed as descriptive rather than inferential; notably, lower baseline voice sound level likely reflects room-to-improve, which motivated excluding baseline domain measures from the primary RF model.

### Relation to prior work

To our knowledge, this is the first study to identify baseline predictors of response to a behavioral speech intervention in PD; most prior prognostic work has addressed subthalamic nucleus deep brain stimulation or non-speech exercise. Our behavioral findings differ from surgical prognostics in deep brain stimulation,^19^ where worse baseline speech and greater motor gains predicted worsening in one year. Differences in mechanism (stimulation trade-offs vs practice-based plasticity), time scale (weeks vs years), and outcomes (UPDRS item vs acoustic/perceptual criteria) likely explain the divergence. Consistent with HiBalance responder work,^20^ only models targeting the trained function exceeded chance and a room-to-improve pattern emerged.

### Interpretation

In the parent RCT we observed clinically relevant gains in voice sound level and small improvements in AVQI;^4^ however, the mean AVQI change did not exceed the reliable change/measurement-error threshold,^26^ so evidence for group-level voice quality change is equivocal. Against that backdrop, the present responder analysis strengthens the case for loudness as a tractable target: individuals presenting with noticeable hypophonia and degraded overall speech impression appear primed to benefit from a high-intensity, cognitively engaging program. The observed plateaus in the partial-dependence curves are consistent with nonlinear, saturating gains - once a minimum impairment is present, mechanisms such as loudness calibration, cueing, and attentional effort may be more effectively engaged. The AVQI pattern indicates that baseline dysphonia does not preclude, and may even predict, intensity gains, but the lack of a robust voice quality model without baseline AVQI cautions against expecting voice quality change from HiCommunication. Notably, the RCT also showed improvements in the perceptual parameter loudness decay (which was not among the RF variables here), further converging on intensity-related benefit. These findings align with accumulated evidence that high-effort speech interventions reliably increase vocal loudness in PD.^6–8^ Lower response probability in the indeterminate motor subtype is plausible if less stable motor profiles hinder cueing and generalization. An alternative, nonexclusive explanation is that “indeterminate” classification can also reflect an earlier disease stage or a less pronounced motor phenotype, making subtype assignment harder; if so, a lighter overall symptom burden could partly account for lower response probability. This is consistent with our observation that pronounced hypophonia and a degraded global speech impression tracked with voice intensity gains, even though severity in loudness is not necessarily correlated with broader motor severity on an individual level. Beyond motor phenotypes, emerging work suggests that PD also exhibits distinct speech subtypes with differential response to levodopa therapy,^38^ underscoring heterogeneity relevant to prognostic research. Finally, LEDD should be interpreted as contextual rather than a modifiable treatment target, as dose reflects disease burden and clinical choices. Given that these findings derive from a single, modestly sized study, clinical use should be cautious and provisional: pre-intervention perceptual ratings of reduced loudness and general impression may help screen for likely voice intensity responders, but thresholds around ∼200–220 VAS and AVQI ≳ 3.5 are hypothesis-generating, not prescriptive. Motor phenotype and LEDD can inform expectation-setting, yet neither should serve as a gatekeeper. Replication in larger, independent samples is needed before incorporating these cues into routine triage or individual decision-making.

Although a small proportion of participants in the active control group met responder criteria, such changes may reflect non-specific effects of structured group training, including social engagement, increased general activation, or exercise-related influences on respiratory–phonatory support. The substantially lower responder proportion compared to HiCommunication, and the limited predictive performance observed in control models, nevertheless suggest that responsiveness was primarily intervention-specific rather than reflecting spontaneous fluctuation alone.

### Methodological considerations

Participants generally presented with mild speech impairment, which limits generalizability beyond similar clinical profiles. Although intelligibility was assessed in the parent trial, ceiling effects limited its utility as a predictive variable. This likely reflects the mild dysarthria severity in the present cohort and may differ in populations with more advanced speech impairment. RF suited the heterogeneous, potentially nonlinear predictor space and enabled permutation importance and partial dependence. Nonetheless, permutation importance can be biased by differences in scale/cardinality, correlated predictors can destabilize ranks, and partial dependence plots show marginal - not causal - effects that may mask interactions. The HiCommunication cohort was modest in size (n = 35), which may increase uncertainty in variable importance rankings and in the shape of partial dependence curves. Although RF models are relatively robust to overfitting, and performance was estimated using OOB predictions, findings should be considered exploratory and require replication in larger samples. Completer-only analyses further limits generalizability. Missingness was low and imputed with missForest, but any imputation can introduce error.

Importantly, our responder thresholds were pragmatic rather than anchored to minimal clinically important differences, which are not established for these measures. The voice intensity threshold (≥2 dB) reflects typical Parkinson–control differences, and the voice quality threshold (AVQI reduction ≥0.54) reflects reliable change/measurement error rather than patient-perceived benefit. Consequently, model performance and inferred thresholds from partial-dependence curves should be viewed as descriptive and may shift with alternative cut-points. Future work should establish minimal clinically important differences for acoustic and perceptual metrics and assess robustness across threshold choices.

### Future directions

Evidence on predictors for behavioral speech interventions in PD remains limited. Generalizability should be examined across delivery formats (individual vs group) and targets (loudness, articulation, prosody, respiratory–phonatory support). Priorities include adequately powered, multi-site replication; harmonized responder criteria and core outcome sets spanning acoustic, perceptual, and communicative participation measures; nested cross-validation and probability calibration; comparative models and interaction-aware explanations; analysis of dose–response and practice adherence; and prospective triage studies where baseline screens inform progression rules.

In the parent trial, group-level improvements were not sustained at the six-month follow-up, suggesting that benefits may attenuate without continued practice or booster sessions. Accordingly, future prognostic research should also examine predictors of durability, not only immediate response. The present responder analysis did not evaluate longer-term prediction due to limited follow-up data in completers.

### Conclusions and clinical implications

Baseline perceptual ratings (reduced loudness, general impression of speech deviation), AVQI, motor subtype, and LEDD jointly predicted improvement in voice intensity following HiCommunication with moderate accuracy, whereas models targeting voice quality and those applied to the active control intervention showed limited performance. While confirmation in larger samples is needed before broader clinical use, the findings suggest that clinically accessible baseline measures may contribute to early expectation-setting, individualized intervention planning, and resource allocation, pending replication in larger cohorts.

## Supplemental Material

sj-docx-1-pkn-10.1177_1877718X261453191 - Supplemental material for Baseline predictors of response to a group-based speech and communication intervention in Parkinson's disease: A secondary analysis of a randomized controlled trial Supplemental material, sj-docx-1-pkn-10.1177_1877718X261453191 for Baseline predictors of response to a group-based speech and communication intervention in Parkinson's disease: A secondary analysis of a randomized controlled trial by Hanna Steurer, Ellika Schalling, Erika Franzén, Joakim Körner Gustafsson and Franziska Albrecht in Journal of Parkinson's Disease
