# Supplementary material for: Baseline predictors of response to a group-based speech and communication intervention in Parkinson's disease: A secondary analysis of a randomized controlled trial
Source: J Parkinsons Dis. 2026 May 25:1877718X261453191. Online ahead of print. doi: 10.1177/1877718X261453191 (PMC13435197; doi:10.1177/1877718X261453191)
Supplement: Supplementary file 1 [file sj-docx-1-pkn-10.1177_1877718X261453191.docx]

Supplement to: Baseline predictors of response to a group-based speech and communication intervention in Parkinson’s disease: a secondary analysis of a randomized controlled trial

Innehåll

[**Random Forest hyperparameters and tuning process**	2](#_Toc223332951)

[**Table S1.** Demographics of the participants in the active control intervention group divided into voice intensity responders and -non-responders.	3](#_Toc223332952)

[**Table S2.** Demographics of the participants in the active control intervention group divided into voice quality responders and -non-responders.	5](#_Toc223332953)

[**Table S3.** Prediction metrics for classification of intervention response using different response domains and including baseline voice sound level and AVQI in their respective models. The table summarizes confusion matrix values and derived metrics including Cohen’s Kappa, precision, recall (sensitivity), specificity, and Youden’s index for each model. These metrics reflect the agreement between predicted and observed intervention response (responder vs. non-responder).	7](#_Toc223332954)

[**Table S4.** Permutation importance (mean decrease in accuracy) for the HiCommunication voice intensity model (baseline voice sound level included)	8](#_Toc223332955)

[**Table S5.** Permutation importance (mean decrease in accuracy) for the HiCommunication voice quality model (baseline AVQI included)	9](#_Toc223332956)

# **Random Forest hyperparameters and tuning process**

Random Forest classifiers were fit using the randomForest package in R. We grew 20,000 trees per model to stabilize out-of-bag (OOB) performance estimates and permutation-based variable importance. At each split, the number of candidate predictors was set to mtry = 4 (approximately √p, where p = 19 predictors), and the minimum terminal node size was set to 7. Hyperparameter checks were conducted using a grid search (mtry 2–10; node size 2–9; sample fraction 0.55–0.80) and tuning functions (tuneRF/tuneRanger), indicating stable OOB error across a range of settings; we therefore retained node size = 7 for all primary models with mtry = 4 för voice intensity models and mtry = 5 for voice quality models. Model performance metrics (κ, precision, recall, specificity, Youden’s index) were calculated from OOB predictions**.**

# **Table S1.** Demographics of the participants in the active control intervention group divided into voice intensity responders and -non-responders.

| Variable | NR (n=33) | R (n=5) | Total (n=38) | p-value |
| --- | --- | --- | --- | --- |
| Age, years |  |  |  | 0.745 |
| Median | 69.00 | 71.00 | 69.00 |  |
| IQR | 5.00 | 8.00 | 5.00 |  |
| Sex, n (%) |  |  |  | 0.067 |
| Male | 19 (57.6%) | 5 (100.0%) | 24 (63.2%) |  |
| Female | 14 (42.4%) | 0 (0.0%) | 14 (36.8%) |  |
| Cognitive subtype, n (%) |  |  |  | 0.636 |
| Non-MCI | 23 (69.7%) | 4 (80.0%) | 27 (71.1%) |  |
| MCI | 10 (30.3%) | 1 (20.0%) | 11 (28.9%) |  |
| Motor subtype, n (%) |  |  |  | 0.100 |
| IND | 5 (15.2%) | 2 (40.0%) | 7 (18.4%) |  |
| PIGD | 23 (69.7%) | 1 (20.0%) | 24 (63.2%) |  |
| TD | 5 (15.2%) | 2 (40.0%) | 7 (18.4%) |  |
| LEDD, mg |  |  |  | 0.846 |
| Median | 600.00 | 500.00 | 551.00 |  |
| IQR | 540.00 | 598.00 | 583.25 |  |
| MDS-UPDRS Total |  |  |  | 0.299 |
| Median | 15.00 | 11.00 | 14.50 |  |
| IQR | 11.00 | 6.00 | 10.50 |  |
| Steps per day |  |  |  | 0.802 |
| Median | 5106.57 | 5331.66 | 5187.01 |  |
| IQR | 3524.36 | 1533.57 | 3447.16 |  |
| Dysarthria test total score |  |  |  | 0.462 |
| Median | 0.09 | 0.07 | 0.08 |  |
| IQR | 0.19 | 0.05 | 0.19 |  |
| Questionnaire of Acquired Speech Disorders |  |  |  | 0.243 |
| Median | 0.67 | 0.03 | 0.63 |  |
| IQR | 0.50 | 0.86 | 0.52 |  |
| Voice sound level |  |  |  | 0.933 |
| Median | 71.36 | 70.67 | 71.33 |  |
| IQR | 6.28 | 0.84 | 5.91 |  |
| Voice sound level Δ |  |  |  | <0.001 |
| Median | -0.01 | 3.09 | 0.38 |  |
| IQR | 2.79 | 1.32 | 2.80 |  |
| AVQI |  |  |  | 0.476 |
| Median | 3.94 | 4.22 | 3.97 |  |
| IQR | 0.65 | 1.04 | 0.73 |  |
| DDK AMR |  |  |  | 0.271 |
| Median | 6.13 | 6.86 | 6.16 |  |
| IQR | 1.40 | 1.15 | 1.42 |  |
| DDK SMR |  |  |  | 0.863 |
| Median | 5.33 | 5.44 | 5.38 |  |
| IQR | 1.29 | 0.50 | 1.22 |  |
| General impression of speech deviation |  |  |  | 0.779 |
| Median | 253.00 | 212.70 | 251.50 |  |
| IQR | 177.00 | 331.00 | 197.75 |  |
| Reduced loudness |  |  |  | 0.778 |
| Median | 177.00 | 210.88 | 205.44 |  |
| IQR | 239.00 | 111.00 | 238.00 |  |
| Harshness |  |  |  | 0.809 |
| Median | 180.00 | 36.00 | 172.00 |  |
| IQR | 316.00 | 301.00 | 312.25 |  |
| HADS Anxiety |  |  |  | 0.118 |
| Median | 6.00 | 3.00 | 5.50 |  |
| IQR | 5.00 | 2.00 | 5.00 |  |
| HADS Depression |  |  |  | 0.368 |
| Median | 4.00 | 2.00 | 3.50 |  |
| IQR | 4.00 | 2.00 | 3.75 |  |
| Verbal fluency phonological |  |  |  | 0.545 |
| Median | 49.00 | 42.00 | 46.00 |  |
| IQR | 25.00 | 4.00 | 20.25 |  |
| Verbal fluency semantic |  |  |  | 1.000 |
| Median | 39.00 | 40.00 | 39.00 |  |
| IQR | 20.00 | 16.00 | 19.25 |  |
| Presence intervention, % |  |  |  | 0.861 |
| Median | 85.00 | 90.00 | 85.00 |  |
| IQR | 10.00 | 5.00 | 10.00 |  |

Note: Values are median (IQR) unless otherwise indicated. p-values are unadjusted (Mann–Whitney U for continuous variables; χ² for categorical variables). All variables were derived at baseline unless labelled with Δ (= post – pre). Abbreviations and variable definitions: AVQI, Acoustic Voice Quality Index (0–10; higher = worse dysphonia); DDK-AMR, Diadochokinetic Alternating Motion Rate (/pa/), syllables/s; DDK-SMR, Diadochokinetic Sequential Motion Rate (/pa-ta-ka/), syllables/s; HADS, Hospital Anxiety and Depression Scale subscales (0–21; higher = worse); IND, indeterminate motor subtype (per Stebbins et al.); IQR, interquartile range; LEDD, levodopa equivalent daily dose (mg); MCI, mild cognitive impairment (per Litvan et al.); MDS-UPDRS, Movement Disorder Society Unified Parkinson’s Disease Rating Scale (higher = worse); NR, non-responder; PIGD, postural instability and gait disorder motor subtype; R, responder; TD, tremor-dominant subtype; Voice sound level, C-weighted SPL during text reading (dBC; higher = louder); Perceptual ratings (general impression of speech deviation, reduced loudness, harshness), clinician VAS 0–1000 (higher = more deviant/greater impairment); Steps per day, 7-day hip-worn accelerometry average; Presence intervention, percentage of attended sessions.

# **Table S2.** Demographics of the participants in the active control intervention group divided into voice quality responders and -non-responders.

| Variable | NR (n=30) | R (n=8) | Total (n=38) | p-value |
| --- | --- | --- | --- | --- |
| Age, years |  |  |  | 0.315 |
| -Median | 69.50 | 68.00 | 69.00 |  |
| IQR | 6.00 | 4.00 | 5.00 |  |
| Sex, n (%) |  |  |  | 0.965 |
| Male | 19 (63.3%) | 5 (62.5%) | 24 (63.2%) |  |
| Female | 11 (36.7%) | 3 (37.5%) | 14 (36.8%) |  |
| Cognitive subtype, n (%) |  |  |  | 0.782 |
| Non-MCI | 21 (70.0%) | 6 (75.0%) | 27 (71.1%) |  |
| MCI | 9 (30.0%) | 2 (25.0%) | 11 (28.9%) |  |
| Motor subtype, n (%) |  |  |  | 0.290 |
| IND | 6 (20.0%) | 1 (12.5%) | 7 (18.4%) |  |
| PIGD | 20 (66.7%) | 4 (50.0%) | 24 (63.2%) |  |
| TD | 4 (13.3%) | 3 (37.5%) | 7 (18.4%) |  |
| LEDD, mg |  |  |  | 0.342 |
| Median | 617.50 | 451.00 | 551.00 |  |
| IQR | 660.12 | 302.50 | 583.25 |  |
| MDS-UPDRS Total |  |  |  | 0.298 |
| Median | 15.50 | 11.50 | 14.50 |  |
| IQR | 10.75 | 7.25 | 10.50 |  |
| Steps per day |  |  |  | 0.562 |
| Median | 5063.64 | 5477.94 | 5187.01 |  |
| IQR | 3248.64 | 3463.45 | 3447.16 |  |
| Dysarthria test total score |  |  |  | 0.706 |
| Median | 0.08 | 0.13 | 0.08 |  |
| IQR | 0.19 | 0.14 | 0.19 |  |
| Questionnaire of Acquired Speech Disorders |  |  |  | 0.062 |
| Median | 0.69 | 0.37 | 0.63 |  |
| IQR | 0.51 | 0.50 | 0.52 |  |
| Voice sound level |  |  |  | 0.470 |
| Median | 71.42 | 70.25 | 71.33 |  |
| IQR | 6.19 | 4.89 | 5.91 |  |
| AVQI Δ |  |  |  | <0.001 |
| Median | 0.33 | -1.14 | 0.05 |  |
| IQR | 0.75 | 0.70 | 0.88 |  |
| AVQI |  |  |  | 0.004 |
| Median | 3.88 | 4.89 | 3.97 |  |
| IQR | 0.60 | 1.00 | 0.73 |  |
| DDK AMR |  |  |  | 0.680 |
| Median | 6.16 | 6.10 | 6.16 |  |
| IQR | 1.44 | 1.33 | 1.42 |  |
| DDK SMR |  |  |  | 0.191 |
| Median | 5.21 | 5.86 | 5.38 |  |
| IQR | 1.14 | 1.00 | 1.22 |  |
| General impression of speech deviation |  |  |  | 0.858 |
| Median | 253.00 | 213.50 | 251.50 |  |
| IQR | 184.25 | 221.00 | 197.75 |  |
| Reduced loudness |  |  |  | 0.471 |
| Median | 225.94 | 134.00 | 205.44 |  |
| IQR | 242.00 | 189.75 | 238.00 |  |
| Harshness |  |  |  | 0.156 |
| Median | 129.50 | 239.00 | 172.00 |  |
| IQR | 291.75 | 160.25 | 312.25 |  |
| HADS Anxiety |  |  |  | 0.801 |
| Median | 6.00 | 5.00 | 5.50 |  |
| IQR | 5.00 | 3.50 | 5.00 |  |
| HADS Depression |  |  |  | 0.884 |
| Median | 3.50 | 3.50 | 3.50 |  |
| IQR | 3.00 | 4.50 | 3.75 |  |
| Verbal fluency phonological |  |  |  | 0.858 |
| Median | 45.00 | 51.00 | 46.00 |  |
| IQR | 17.50 | 23.50 | 20.25 |  |
| Verbal fluency semantic |  |  |  | 0.351 |
| Median | 39.00 | 48.50 | 39.00 |  |
| IQR | 15.75 | 18.00 | 19.25 |  |
| Presence intervention, % |  |  |  | 0.585 |
| Median | 85.00 | 90.00 | 85.00 |  |
| IQR | 10.00 | 26.25 | 10.00 |  |

Note: Values are median (IQR) unless otherwise indicated. p-values are unadjusted (Mann–Whitney U for continuous variables; χ² for categorical variables). All variables were derived at baseline unless labelled with Δ (= post – pre). Abbreviations and variable definitions: AVQI, Acoustic Voice Quality Index (0–10; higher = worse dysphonia); DDK-AMR, Diadochokinetic Alternating Motion Rate (/pa/), syllables/s; DDK-SMR, Diadochokinetic Sequential Motion Rate (/pa-ta-ka/), syllables/s; HADS, Hospital Anxiety and Depression Scale subscales (0–21; higher = worse); IND, indeterminate motor subtype (per Stebbins et al.); IQR, interquartile range; LEDD, levodopa equivalent daily dose (mg); MCI, mild cognitive impairment (per Litvan et al.); MDS-UPDRS, Movement Disorder Society Unified Parkinson’s Disease Rating Scale (higher = worse); NR, non-responder; PIGD, postural instability and gait disorder motor subtype; R, responder; TD, tremor-dominant subtype; Voice sound level, C-weighted SPL during text reading (dBC; higher = louder); Perceptual ratings (general impression of speech deviation, reduced loudness, harshness), clinician VAS 0–1000 (higher = more deviant/greater impairment); Steps per day, 7-day hip-worn accelerometry average; Presence intervention, percentage of attended sessions.

# **Table S3.** Prediction metrics for classification of intervention response using different response domains and including baseline voice sound level and AVQI in their respective models. The table summarizes confusion matrix values and derived metrics including Cohen’s Kappa, precision, recall (sensitivity), specificity, and Youden’s index for each model. These metrics reflect the agreement between predicted and observed intervention response (responder vs. non-responder).

| Domain | Group | TN | FP | FN | TP | Precision | Recall | Specificity | Youden’s Index | Cohen’s Kappa | Class. error |
| --- | --- | --- | --- | --- | --- | --- | --- | --- | --- | --- | --- |
| Voice Intensity | HiCommunication | 9 | 5 | 2 | 19 | 0.79 | 0.90 | 0.64 | 0.55 | 0.57 | 0.20 |
| Voice Intensity | Active Control | 33 | 0 | 5 | 0 | NA* | 0.00 | 1.00 | 0.00 | 0.00 | 0.13 |
| Voice Quality | HiCommunication | 18 | 3 | 7 | 7 | 0.70 | 0.5 | 0.86 | 0.36 | 0.38 | 0.29 |
| Voice Quality | Active Control | 29 | 1 | 8 | 0 | 0.00 | 0.00 | 0.97 | -0.03 | -0.05 | 0.24 |
| Note: Precision is undefined (NA) for the active control voice intensity model because no true positives (TP) were predicted. All Cohen’s Kappa values are unweighted. According to Cohen’s guidelines, values of kappa ≤ 0 indicate no agreement, values between 0.01 and 0.20 indicate slight agreement, 0.21–0.40 fair agreement, 0.41–0.60 moderate agreement, 0.61–0.80 substantial agreement, and 0.81–1.00 almost perfect agreement. Classification error is calculated as (FP + FN) divided by the total number of observations (TN + FP + FN + TP), representing the overall proportion of incorrect predictions. Abbreviations: NR = non-responder, R = responder, TN = true negative, FP = false positive, FN = false negative, TP = true positive, NA = not available. | | | | | | | | | | | |

# **Table S4.** Permutation importance (mean decrease in accuracy) for the HiCommunication voice intensity model (baseline voice sound level included)

| Variables | Accuracy NR | Accuracy R | Accuracy all |
| --- | --- | --- | --- |
| DDK AMR | -5,72 | -12,39 | -11,97 |
| Verbal fluency phonological | -9,84 | -9,40 | -11,92 |
| Perceptual harshness | -10,46 | -7,35 | -10,86 |
| HADS depression | -8,53 | -4,99 | -8,24 |
| MDS-UPDRS total | -9,52 | -3,33 | -7,76 |
| HADS anxiety | -7,42 | -4,11 | -6,83 |
| Verbal fluence semantic | -4,97 | -2,68 | -4,74 |
| Sex | -4,44 | -2,76 | -4,10 |
| Age | -6,47 | 0,75 | -3,31 |
| Cognitive subtype | -5,17 | 0,88 | -2,34 |
| DDK SMR | -1,41 | 0,28 | -1,00 |
| Dysarthria test total score | -1,92 | 5,48 | 2,20 |
| Steps per day | -3,75 | 7,40 | 2,33 |
| Questionnaire on Acquired Speech Disorders | -3,37 | 12,22 | 7,44 |
| Voice sound level | 14,24 | 13,78 | 16,99 |
| AVQI | 15,28 | 15,83 | 17,83 |
| LEDD | 20,69 | 9,17 | 18,03 |
| Motor subtype | 10,17 | 23,97 | 21,87 |
| Perceptual general impression of speech deviation | 26,29 | 23,62 | 29,56 |
| Perceptual reduced loudness | 71,91 | 77,62 | 84,14 |

Note: Values show the percent increase in out-of-bag misclassification when the given predictor is randomly permuted. Larger positive numbers indicate greater importance. “Accuracy NR” and “Accuracy R” quantify class-specific importance when evaluating only Non-responders or Responders, respectively; “Accuracy all” is the overall importance across both classes. Predictors are shown in descending order of overall importance. In this model, perceptual reduced loudness and perceptual general impression dominated, followed by motor subtype, levodopa equivalent daily dose, Acoustic Voice Quality Index, and baseline voice sound level. Near-zero or negative values indicate little unique signal (or permutation noise/collinearity). AVQI = Acoustic Voice Quality Index (0–10; higher = worse dysphonia); DDK AMR/SMR = diadochokinetic alternating/sequential motion rate (syllables/s); HADS = Hospital Anxiety and Depression Scale (0–21 per subscale; higher = worse); LEDD = levodopa equivalent daily dose (mg); MDS-UPDRS Total = Movement Disorder Society Unified Parkinson’s Disease Rating Scale motor total (higher = worse); Motor subtype = IND (indeterminate), PIGD (postural instability/gait difficulty), TD (tremor-dominant); NR = Non-responder; R = Responder; Steps/day = 7-day hip-worn accelerometry average; Perceptual ratings (reduced loudness, general impression, harshness) are clinician visual-analogue scales 0–1000 with higher values indicating greater deviation. Mean decrease accuracy was computed via permutation on out-of-bag samples from the fitted random-forest model; class-specific importances were calculated by restricting evaluation to each class. Negative importances can arise when permutation marginally improves accuracy due to sampling variability or correlated predictors and should be interpreted as ≈0.

# **Table S5.** Permutation importance (mean decrease in accuracy) for the HiCommunication voice quality model (baseline AVQI included)

| Variables | Accuracy NR | Accuracy R | Accuracy all |
| --- | --- | --- | --- |
| DDK AMR | -17,12 | -12,91 | -18,31 |
| LEDD | -16,05 | -13,14 | -18,12 |
| HADS anxiety | -11,70 | -9,85 | -13,96 |
| Verbal fluency semantic | -4,69 | -12,99 | -11,34 |
| Motor subtype | -8,83 | -7,18 | -9,92 |
| Perceptual general impression of speech deviation | -11,18 | -3,54 | -9,54 |
| Perceptual harshness | -9,72 | -5,60 | -9,49 |
| Verbal fluency phonological | -6,15 | -7,83 | -8,59 |
| HADS depression | -7,68 | -4,98 | -8,09 |
| Cognitive subtype | -4,87 | -6,76 | -6,82 |
| Age | -8,85 | -0,73 | -5,93 |
| Steps per day | -8,36 | 0,60 | -5,16 |
| Voice sound level | -3,03 | -4,67 | -4,76 |
| Questionnaire on Acquired Speech Disorders | -1,43 | -6,12 | -4,40 |
| Dysarthria test total score | 0,38 | -4,00 | -2,84 |
| MDS-UPDRS total | 3,74 | 4,78 | 5,09 |
| Perceptual reduced loudness | 7,27 | 13,08 | 12,14 |
| DDK SMR | 10,01 | 10,53 | 12,83 |
| AVQI | 79,45 | 82,44 | 91,34 |

Note: Values show the percent increase in out-of-bag misclassification when the given predictor is randomly permuted. Larger positive numbers indicate greater importance. “Accuracy NR” and “Accuracy R” quantify class-specific importance when evaluating only Non-responders or Responders, respectively; “Accuracy all” is the overall importance across both classes. Predictors are shown in descending order of overall importance. In this model, perceptual reduced loudness and perceptual general impression dominated, followed by motor subtype, levodopa equivalent daily dose, Acoustic Voice Quality Index, and baseline voice sound level. Near-zero or negative values indicate little unique signal (or permutation noise/collinearity). AVQI = Acoustic Voice Quality Index (0–10; higher = worse dysphonia); DDK AMR/SMR = diadochokinetic alternating/sequential motion rate (syllables/s); HADS = Hospital Anxiety and Depression Scale (0–21 per subscale; higher = worse); LEDD = levodopa equivalent daily dose (mg); MDS-UPDRS Total = Movement Disorder Society Unified Parkinson’s Disease Rating Scale motor total (higher = worse); Motor subtype = IND (indeterminate), PIGD (postural instability/gait difficulty), TD (tremor-dominant); NR = Non-responder; R = Responder; Steps/day = 7-day hip-worn accelerometry average; Perceptual ratings (reduced loudness, general impression, harshness) are clinician visual-analogue scales 0–1000 with higher values indicating greater deviation. Mean decrease accuracy was computed via permutation on out-of-bag samples from the fitted random-forest model; class-specific importances were calculated by restricting evaluation to each class. Negative importances can arise when permutation marginally improves accuracy due to sampling variability or correlated predictors and should be interpreted as ≈0.
